# A Break from the Norm? Parametric Representations of Preference Heterogeneity for Discrete Choice Models in Health

**DOI:** 10.1177/0272989X251357879

**Published:** 2025-09-05

**Authors:** John Buckell, Alice Wreford, Matthew Quaife, Thomas O. Hancock

**Affiliations:** Nuffield Department of Population Health, University of Oxford, Oxford, UK; University of East Anglia, Norwich, Norfolk, UK; Evidera, London, UK; Choice Modelling Centre, University of Leeds, UK; Nuffield Department of Population Health, University of Oxford, Oxford, UK

**Keywords:** discrete choice experiment, choice model, mixed logit, random parameters logit, model averaging

## Abstract

**Background:**

Any sample of individuals has its own unique distribution of preferences for choices that they make. Discrete choice models try to capture these distributions. Mixed logits are by far the most commonly used choice model in health. Many parametric specifications for these models are available. We test a range of alternative assumptions and model averaging to test if or how model outputs are affected.

**Design:**

Scoping review of current modeling practices. Seven alternative distributions and model averaging over all distributional assumptions were compared on 4 datasets: 2 were stated preference, 1 was revealed preference, and 1 was simulated. Analyses examined model fit, preference distributions, willingness to pay, and forecasting.

**Results:**

Almost universally, using normal distributions is the standard practice in health. Alternative distributional assumptions outperformed standard practice. Preference distributions and the mean willingness to pay varied significantly across specifications and were seldom comparable to those derived from normal distributions. Model averaging offered distributions allowing for greater flexibility and further gains in fit, reproduced underlying distributions in simulations, and mitigated against analyst bias arising from distribution selection. There was no evidence that distributional assumptions affected predictions from models.

**Limitations:**

Our focus was on mixed logit models since these models are the most common in health, although latent class models are also used.

**Conclusions:**

The standard practice of using all normal distributions appears to be an inferior approach for capturing random preference heterogeneity. **Implications.** Researchers should test alternative assumptions to normal distributions in their models.

**Highlights:**

- Health modelers use normal mixing distributions for preference heterogeneity.
- Alternative distributions offer more flexibility and improved model fit.
- Model averaging offers yet more flexibility and improved model fit.
- Distributions and willingness to pay differ substantially across alternatives.

Discrete choice models are used widely across health economics to answer questions on health behaviors and clinical choices, to inform the development of randomized controlled trials, for policy choices and public opinion, and in economic evaluation. Hundreds are published each year.^1^ Policymakers such as the National Institute for Health and Care Excellence^2^ and the Food and Drug Administration^3^ are increasingly using evidence from these methods.

Broadly speaking, there are 3 acknowledged types of heterogeneity in choice behavior: preference heterogeneity (differences in preferences for attributes and alternatives), scale heterogeneity (differences in the randomness in choices), and decision rule heterogeneity (the decision rules/heuristics that individuals use when making choices). In health economics, attention has been given to scale heterogeneity^4,5^ and to decision rule heterogeneity,^6–8^ but the overwhelming attention has been in attempting to capture preference heterogeneity.^9^ Here, standard practices have emerged over the past 2 decades, which we bring into question.

Preference heterogeneity can either be classed as deterministic or random. Deterministic preference heterogeneity involves relating choice behavior to observed individual characteristics. Random preference heterogeneity is that which is unobserved to the analyst and can be modeled in a variety of ways, broadly divided into discrete and continuous mixtures (see Figure 1). In this article, we focus on the specification of mixing distributions in continuous mixture models, which are the most frequently implemented choice model in health economics. As per Figure 1, there are many other approaches that researchers could adopt, including allowing for correlation across mixing distributions,^10^ latent classes,^11^ mixed latent class models,^12^ logit mixed logit models,^13^ and nonparametric approaches.^14^ Researchers may combine these structures and/or average over them. In this article, we highlight some approaches and compare them the researcher norms in the field.

**Figure 1:**
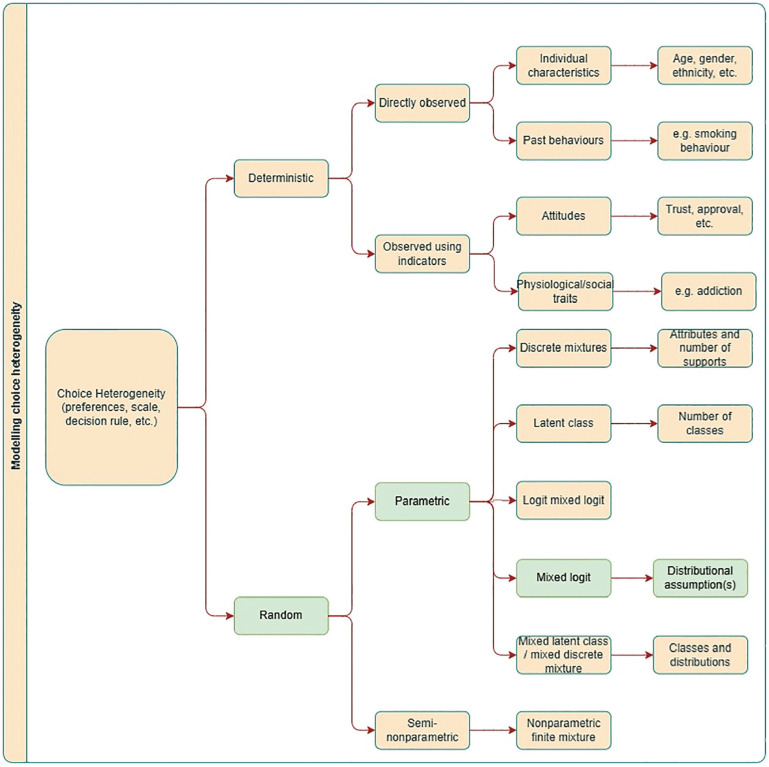
An overview of how heterogeneity can be modeled within discrete-choice models. We specifically focus on the distributional forms assumed within mixed logit models. Note that this list is not exhaustive; other options exist.

Examples of both approaches abound in health, although recent evidence shows that analysts in health are using continuous mixtures more than any other model.^9^ Of these continuous mixtures, recent evidence indicates that studies in health are overwhelmingly assuming normal distributions to model preference heterogeneity.^9^ We extended this analysis with a scoping review assessing current practices of modeling unobserved preference heterogeneity in health-based choice modeling, corroborating these findings.

In recent years, there have been major advances in the way that choice modelers have been able to capture random preference heterogeneity. These include transformations of basic distributions to impose constraints on preferences, such as log-uniform distributions that impose a directionality of preference^15,16^ and more flexible distributions that allow for asymmetry such as asymmetric triangular^17^ or using transformations of normal as in Fosgerau and Mabit.^18^ Further to this is model averaging,^19,20^ which can potentially improve on individual specification and, more importantly, mitigates the risk of analyst bias (for example, observer bias in which the choice of distribution might bias estimates^21^). It can also protect against overfitting, with Hancock et al.^20^ demonstrating that model averaging performs particularly well in forecasting, likely a result of at least 1 constituent model accurately predicting choices made by each individual in the holdout sample.

Two further issues that arise with sole reliance on normal distributions concern willingness to pay (WTP). First, as might be expected, if the normal distribution is not a reasonable depiction of the true preference heterogeneity, then bias can be introduced in the distribution of WTP.^22^ Importantly, even if the research interest is only in the mean, rather than the distribution, of WTP, this too could be biased. This is potentially highly problematic if results from these studies are used elsewhere (e.g., in cost–benefit analysis). Second, normal distributions can prevent moments of marginal WTP distributions,^23^ and alternative distributions or indeed specifications are used elsewhere for estimating this instead.^24,25^

We use 4 datasets in analyses to examine alternative specifications of continuous mixing distributions. Two are stated preferences: smoking choices in the United States and HIV prevention choices in South Africa. These were chosen due to known preference heterogeneity. For example, it is well known that the preference for menthol flavors varies among smoker groups, implying that the preference distribution is likely asymmetric.^26^ A further dataset is a revealed preference analysis of smoking and vaping in the United States. This was chosen to demonstrate the applicability of these methods in modeling real-world choices and to extend the modeling framework from the multinomial logit to multivariate logit models with correlated errors. A simulated dataset of drug choices completes the set. An advantage is that these data can be shared, along with the code script, for replicability and use by researchers in the field.

Using these datasets, we compare standard practice with alternative representations of continuous mixing distributions. More specifically, we examine model fit, choice probabilities, WTP means, and preference distributions. We reconcile our scoping review with our empirical exercises to ask whether current practices can be improved and the extent to which this is important for empirical measures derived from choice models in health. By comparing standard practices with more advanced approaches, we provide evidence on the robustness of modeling results in health-based choice modeling.

The remainder of this article is structured as follows. In the next section, we present our scoping review. We then present the datasets used in exercises. Next, we set out our choice modeling methods and strategy for making model comparisons. We then move to the modeling results. Finally, we discuss our findings and present our conclusions.

## Methods

### Sampling and Datasets

#### Smokers’ stated preference tobacco product choice data, United States

Data were taken from an online discrete-choice experiment (DCE) on 2,031 US adult smokers conducted in 2017 (1,531 current smokers and 500 self-reported recent quitters).^26^ Sampling was based on quotas derived from the Behavioural Risk Factor Surveillance System data in 2013–14, comprising gender, age, education, and region to make the sample representative. The sample size is well in excess of minimum sample size calculations.^27^ A series of exercises was conducted to promote the quality of the data (e.g., attention checks in the survey, minimum time threshold, and removing duplicate individuals).

The DCE was based on a review of the literature and a pilot study. The literature review comprised prior DCEs in tobacco,^28^ market data on tobacco product prices,^29^ and scientific literature on the harms of tobacco products.^30^ In the study, individuals chose between cigarettes, e-cigarettes, and opt-outs. Respondents were presented with 2 of each product and made 2 choices in each choice task. Attributes (levels) were price ($4.99, $7.99, $10.99, $13.99), flavors (tobacco, menthol, fruit, sweet), level of nicotine (none, low, medium, high), and health harm expressed in life-years lost to the average smoker (2 y, 5 y, 10 y, unknown). Some levels were omitted to make choices realistic (e.g., fruit/sweet cigarettes are not on the market in the United States). This design is based on reality (nonmenthol tobacco flavors being banned), a review of the literature, and a pilot study; full details can be found in Buckell et al.^26^

A Bayesian D-optimal design was used.^31^ Priors were obtained from a multinomial logistic (MNL) model in an analysis of pilot study data on 87 respondents. Three blocks of 12 had individuals randomized to them. Each individual answered 12 choice sets, balancing concerns of learning and respondent fatigue.^32^ A practice choice scenario was given to all respondents to ensure that they understood how the choice scenarios worked.

#### Smokers’ revealed preference tobacco product choice data, United States

Smoking behavior data were collected from the 2,031 sampled individuals. Each was asked about their use of cigarette and e-cigarettes. A total of 1,038 reported cigarette use only, 148 reported e-cigarette use only, and 619 reported the use of both products; 226 reported that they had recently quit. Data on individuals’ characteristics were also collected.

#### HIV prevention stated preference product choices among a general population sample, South Africa

In 2015, 367 HIV-negative women (199 aged 16–17 y and 168 aged 18–49 y) were interviewed in a randomized face-to-face household survey conducted in a periurban township on the outskirts of Johannesburg, South Africa.^33^

The DCE was developed through an analysis of a previous DCE and focus groups discussions carried out in previous research,^34^ specifically identifying important characteristics of prevention products and exploring optimal ways to present these in a clear and relatable manner to participants. This was supplemented by a scoping literature review to identify new products and additional attributes that could be important to respondents, which was added to and refined through piloting. Three alternatives of new products were shown in each task using an unlabeled design in which each alternative represents a generic product within which all characteristics can change as prescribed by the statistical design. In this experiment, respondents chose between 3 unlabeled alternatives of new HIV prevention products and an opt-out. Products were described by product type (oral pill, injectable, reusable diaphragm, vaginal gel, and vaginal ring), HIV prevention efficacy (55%, 75%, 95%), contraceptive ability (yes, no), sexually transmitted infection protection (yes, no), frequency of use (coitally, daily, weekly, monthly, every 3 mo, every 6 mo, annually), and side effects (nausea, stomach cramps, dizziness, none). A Bayesian D-optimal design was generated using priors estimated on an MNL model in a pilot using a sequential orthogonal design.

#### Simulated drug choices data

Simulated drug choice data were generated for 1,000 individuals, each of whom completed 10 choice tasks. In each task, 2 branded alternatives and 2 unbranded alternatives were presented, described by country of production, characteristics of the drug (standard, fast acting, or double strength), risk of side effects, and price. The attribute levels were based on the example choice dataset given on the Apollo choice modeling website. The choices were generated using random draws U[0,1], with the probability of choosing each alternative generated using an MNL model. The utility for each alternative was defined by specifying a taste for each attribute for each individual (drawn from distributions), where different underlying distributions were used for different attributes.

### Choice Modeling

Random utility maximization (RUM) models have been used almost exclusively for choice models in health.^7,8^ In this formulation, the individual reconciles their product/attribute preferences for each of the available alternatives and chooses that which maximizes their utility. Respondents’ utility is typically specified by the modeler as a linearly additive function of attribute/alternative preferences and the alternative attribute combinations available, with an error term to capture noise. For each alternative, the individual is assumed to choose the option that delivers the highest utility.

(1) $$U_{nti}=V_{nti}+\varepsilon_{nti},$$

where $U_{nti}$ is the utility for decision maker *n* for alternative *i* in choice task *t*, comprising deterministic and random utility. $V_{nti}$ is the deterministic component of utility, and $\varepsilon_{nti}$ is the random component of utility,^35^ capturing the fact that the analyst does not observe all factors that may lead to a decision, where factors may vary across individuals, alternatives, or specific choice tasks.

For the revealed preference data, logit models are specified on both outcomes.

(2) $$U_{cig,n}=V_{cig,n}+\rho_{n}+\varepsilon_{cig,n}$$

and

(3) $$U_{ecig,n}=V_{ecig,n}+\rho_{n}+\varepsilon_{ecig,n},$$

where $V_{cig,n}$ is the deterministic component of utility for cigarette use and $V_{ecig,n}$ is the deterministic component of utility for e-cigarette use. $\rho_{n}$ is an individual-specific error component capturing the correlation across the errors for both outcomes. This captures the correlation across product use such that a positive estimate means those who use cigarettes are also more likely to use e-cigarettes and vice versa for a negative estimate. It does not measure relative utility for these products (see a later section). $\rho_{n}$ here assumes a positive correlation; to test for a negative correlation, we simply replace $\rho_{n}$ with $-\rho_{n}$ in (3). This did not improve model performance; hence, we retained the original specification.

### Choice Probabilities

For stated preference and simulated data, the RUM model is operationalized by assuming a type I extreme value error distribution on $\varepsilon_{nti}$ for each alternative and estimating choice probabilities for each alternative, resulting in the MNL model.

(4) $$P_{nti}=\frac{\exp(V_{nti})}{\sum_{j=1\ldots J}\exp(V_{\mathrm{ntj}})}.$$

where $P_{nti}$ is the RUM probability of respondent *n* choosing alternative *i* from set *J* in a choice task. The probability of individual *n* making a sequence of choices, each *t*, over the total, $T_{n}$ , choice tasks that they face is then

(5) $$P_{n}=\overset{T_{n}}{\underset{t=1}{\Pi }}P_{ntj^{*}},$$

where $j^{*}$ is the alternative selected in each given scenario.

For the revealed preference data, assuming a type I extreme value error distribution on $\varepsilon_{n,cig}$ and $\varepsilon_{n,cig}$ gives

(6) $$P_{n,cig}=\frac{\exp(V_{n,cig}+\rho_{n})}{1+\exp(V_{n,cig}+\rho_{n})}$$

and

(7) $$P_{n,ecig}=\frac{\exp(V_{n,ecig}+\rho_{n})}{1+\exp(V_{n,ecig}+\rho_{n})},$$

where $P_{n,cig}$ is the probability of reported cigarette use and $P_{n,ecig}$ is the probability of reported e-cigarette use. Thus, we have

(8) $$P_{n}={\left(P_{n,cig}\right)}^{c_{n}}.{\left(1-P_{n,cig}\right)}^{\left(1-c_{n}\right)}.{\left(P_{n,ecig}\right)}^{e_{n}}.{\left(P_{n,ecig}\right)}^{\left(1-e_{n}\right)},$$

where $c_{n}$ and $e_{n}$ are dummy variables that take a value of 1 if individual *n* is a smoker or e-cigarette smoker, respectively.

Model log-likelihoods are given by

(9) $$LL=\sum_{n=1}^{N}\ln\;P_{n}.$$

### Model Averaging

The approach here follows the sequential latent class approach.^20,21^ This formulation adapts a standard latent class framework such that the classes are the set of models over which model averaging occurs, and the class membership probability is the weight applied to each model. The constituent models—each treated as a class in the LC model—are estimated separately in a prior estimation stage. They are then entered into the latent class framework with the parameter estimates for the models held constant while the class shares are estimated. Specifically, supposing there are *K* models, each has a set of estimated parameters that we denote ${\hat{\Omega }}_{k}$ . Then, the only parameters to be estimated, $\theta_{k}$ , are those that feed the class membership probabilities, $\pi_{k}$ , which is the averaging. That is, the estimated class membership probability parameters govern the weights for each constituent model and optimize model fit. Thus, the model averaging will give more weight to constituent models with superior fit.

(10) $$\pi_{k}=\frac{\exp(\theta_{k})}{\sum_{k=1}^{K}\exp(\theta_{k})}$$

where the logistic form ensures $\sum_{k=1}^{K}\pi_{k}=1$ and $0\leq \pi_{k}\leq 1\;\forall k$ .

Combining the components leads to the log-likelihood for sequential latent class model averaging,

(11) $$LL_{MA}\left(\pi_{k},{\hat{\Omega }}_{k}\right)=\overset{N}{\underset{n=1}{\Pi }}\ln\left(\sum_{k=1}^{K}\pi_{k}\cdot P_{n}\left({\hat{\Omega }}_{k}\right)\right)$$

Although it is possible to average over any set of models, we restrict our modeling in this setting to 3 groups of models, and our presentation to one group containing all constituent models as that yielded the largest gain in fit and flexibility. The groups of models were the base models MA(S,U,T), base + extended models (S,U,T,LN, LU), and base + extended + flexible models MA(S,U, T,LN,LU,FM2,FM3).

### Application of Choice Models to Datasets

The deterministic component(s) of utility is then defined for each dataset. For tobacco stated preference data,

(12) $$\begin{array}{c} V_{nti}=ASC_{cig,n}.{Cig}_{nti}+ASC_{ecig,n}.{Ecig}_{nti}\\ +\beta_{p,n}.{Price}_{nti}+\beta_{N,n}.{Nicotine}_{nti}\\ +\beta_{f,n}.{Flavor}_{nti}+\beta_{h,n}.{Heath\;Harm}_{nti} \end{array}$$

where $V_{nti}$ includes alternative-specific constant terms that are added if alternative *i* is a cigarette ( $ASC_{cig,n}$ ) or an e-cigarettes ( $ASC_{ecig,n}$ ), where the opt-out is the reference product. We then have attributes of price, nicotine, flavors, and health harm expressed in the number of life years lost; and corresponding preferences ( β ) which vary over individuals, *n*, according to distributions (cf. Table 1). This model is also estimated in the WTP space,^24^ which avoids undefined moments in the WTP distribution.^23^

(12a) $$\begin{array}{c} V_{nti}=\beta_{p,n}.({Price}_{nti}+ASC_{cig,n}.{Cig}_{nti}\\ +ASC_{ecig,n}.{Ecig}_{nti}+\beta_{N,n}.{Nicotine}_{nti}\\ +\beta_{f,n}.{Flavor}_{nti}+\beta_{h,n}.{Heath\;Harm}_{nti}) \end{array}$$

**Table 1:** Specifications of Mixing Distributions^a^

Distribution Name	Form	Implementation	Symmetrical	Unidirectional	Bounded Support
Normal	$\tau_{m}\sim N(\mu_{m},\sigma_{\tau ,m}^{2})$	$\beta_{m}=\;\mu_{m}+\;\sigma_{m}*d_{N,m}$	Yes	No	No
Uniform	$\tau_{m}\sim U[a_{m},a_{m}+b_{m}]$	$\beta_{m}=\;a_{m}+\;b_{m}*d_{U,m}$	Yes	No	Yes
Triangular	$\tau_{m}\sim T[a_{m},a_{m}+b_{m}]$	$\beta_{m}=\;a_{m}+\;b_{m}*(d_{U1,m}+d_{U2,m})$	Yes	No	Yes
Log normal	$\tau_{m}\sim \mathrm{LN}(\mu_{m},\sigma_{\tau ,m}^{2})$	$\beta_{m}=\;-e^{\left(\mu_{m}+\;\sigma_{m}*d_{N,m}\right)}$	No	Yes	No
Log uniform	$\tau_{m}\sim \mathrm{LU}[a_{m},a_{m}+b_{m}]$	$\beta_{m}=\;-e^{\left(a_{m}+\;b_{m}*d_{U,m}\right)}$	No	Yes	Yes
Asymmetric triangular	$\tau_{m}\sim \mathrm{AT}[a_{m},b_{m},c_{m}]$	$\beta_{m,\mathrm{lower}}=a_{m}+(((a_{m}+b_{m}/2)+c_{m})-a_{m})*\sqrt{d_{U1,m}}$ $\beta_{m,\mathrm{upper}}=b_{m}-(b_{m}-((a_{m}+b_{m}/2)+c_{m}))*\sqrt{d_{U2,m}}$	No	No	Yes
Fosgerau and Mabit	$\tau_{m}\sim \sum_{p=0}^{P}\alpha_{p,m}u^{p,m}$	$\beta_{m}=\mu_{m}+\sum_{p=0}^{P}\sigma_{p,m}*d^{p,m}$	No	No	No

$\mu_{m}$ is an estimated mean of a distribution; $\sigma_{m}$ is an estimated standard deviation of a distribution; $a_{m}$ is an estimated bound of a distribution; $b_{m}$ is an estimated range of a distribution; $c_{m}$ is an estimated offset; $d_{N,m}$ are draws from a standard (i.e., *N*(0,1)) normal distribution; $d_{U,m}$ are draws from a standard (i.e., *U*[0,1]) uniform distribution. In all cases, 500 modified Latin hypercube sampling^37^ draws are taken. Estimation of the asymmetric triangular follows the procedure set out in Dekker,^17^ where to reduce model runtime, $c_{m}$ is fixed to a value of 0 for an attribute if its inclusion does not significantly improve model fit. Note that for the applications in this work, tastes are assumed to vary across individuals, *n*. However, preferences may also vary across choice context, *t*, if we were to allow for inter- and intrarespondent heterogeneity.^38^

For HIV prevention,

(13) $$\begin{array}{c} V_{nti}=ASC_{optout,n}.{Optout}_{nti}+\beta_{p,n}.Product\;{type}_{nti}\\ +\beta_{pro,n}.Protection\;from\;{diseases}_{nti}\\ +\beta_{pre,n}.{Prevention\;pregnancy}_{nti}\\ +\beta_{freq,n}.Frequency\;of\;{use}_{nti}\\ +\beta_{se,n}.{Side\;effects}_{nti} \end{array}$$

Where $V_{nti}$ includes an alternative-specific constant for the opt-out; and attributes of product type, protection from diseases, pregnancy prevention, frequency of use, and side effects with corresponding preferences ( β ) which vary over individuals, *n*, according to distributions (cf. Table 1).

For simulated drug choices,

(14) $$\begin{array}{c} V_{nti}=\beta_{b,n}.{Branded}_{nti}+\beta_{c,n}.{Country}_{nti}\\ +\beta_{ch,n}.{Characteristic}_{nti}+\beta_{se,n}.{Side\;effects}_{nti}\\ +\beta_{p,n}.{Price}_{nti} \end{array}$$

Where $V_{nti}$ includes attributes of branded, country of origin, drug characteristic (e.g. fast acting), side effects, and price with corresponding preferences ( β ) which vary over individuals, *n*, according to distributions (cf. Table 1).

For tobacco revealed preference,

(15) $$V_{cig,n}=ASC_{cig,n}+\gamma_{cig,n}.z_{n}$$

and

(16) $$V_{ecig,n}=ASC_{ecig,n}+\gamma_{ecig,n}.z_{n},$$

where $V_{n}$ includes an alternative-specific constant for cigarettes/e-cigarettes, which vary over individuals, *n*, according to distributions (cf. Table 1). $z_{n}$ are individual characteristics, with corresponding parameters ( γ) to be estimated. $z_{n}$ can contain cigarette product use to capture the relative preferences for these products but may require correction for endogeneity.^36^

In all specifications, attribute levels are dummy coded (which is equivalent to effects coding, as used widely in health^23^) except for price, which is treated continuously.

The utility functions above can then be extended to accommodate deterministic and random heterogeneity. Given our interest in the latter, our discussion focuses on this. Random heterogeneity can take 2 main forms, namely, discrete mixtures^11^ (i.e., latent classes) and continuous mixtures^35^ (i.e., mixed logit models). Latent classes generally alter all of the coefficients (although they need not), allowing for a number of classes each with their own fixed set of parameter estimates. Mixed logit models specify the preference for some or often all attributes as a distribution, which can take a wide range of forms, a point that we next consider. Mixed latent class models combine both structures, in which a set of classes are estimated and distributions of preferences are specified within each class.

### Distributional Assumptions in Mixed Logit Models

Given the freedom with which researchers can specify mixed logit models, there are an almost infinite number of possible model specifications. Thus, a comparison across all sets of possibilities is unwieldy. To make a reasonable set of models, and then comparisons across models, 3 classes of models are defined in the current work. We begin with what we refer to as a “basic” set, which includes the standard (N) approach in current practice in health; that is, setting all mixing distributions to be normal (see Table 1). We next use 2 further “basic” distributional assumptions that are used in the choice modeling literature (predominantly outside of health research), namely, uniform (U) and triangular (T). We next define a group of “extended” basic distributions, in which we take transformations of 2 basic forms to allow for asymmetry in the distributions of preferences. Here, we have log normal (LN) and log uniform (LU) distributions. LN and LU are desirable specifications for when strictly positive or negative preferences are to be imposed (although in this case, we use only negative transformations). Finally, we use a group of “flexible” distributions that are the asymmetric triangular (AT) and the polynomial expansions set out in Fosgerau and Mabit^18^ to allow for further flexibility. Specifically, we use second-order (FM2) and third-order (FM3) specifications. The mathematical form and implementation of these distributional assumptions are set out in Table 1.

### Model Estimation

All models are estimated using the Apollo package in R.^39^ The models used 500 modified Latin hypercube sampling draws, except for the tobacco RP dataset, which used 100. This was due to the difficulty in estimating these models, where 100 draws made for more stability in estimation (we recognize that this may be too few; see the limitations section). For each model, comparisons are made across the model fit (log-likelihood) and the estimated choice probabilities for each model. Choice probabilities are computed using sample enumeration.^35^ Unconditionals are constructed from the fitted model postestimation and used for analyses of preference/WTP distributions. For model averaging, unconditionals are sampled from each constituent model according to the weights (derived from the estimates of $\theta_{k}$ ). The estimation of model averaging is thus a simple process (it requires only model outputs $P_{n}$ from each model), and the analyst need not know the underlying model that generated $P_{n}.$ ^20^

Codes and simulated data are available on GitHub at https://github.com/johnbuckell/Modelling-random-preference-heterogeneity-in-health-choices.

## Results

### Scoping Review

Table A1 shows the results for types of distributions used in the mixed logit models retrieved in our search. In 2017, 98% (226/230) of all distributions were normal, with 2% (4/230) being log normal. In 2022, 99% (736/746) of all distributions were normal, with 1% (10/746) being log normal. Based on this, we define a “standard practice” in health to be using normal distributions for all parameters. Notably, almost half of papers did not report the distributional assumptions used in their model. This was part of a worrying theme of not reporting essential information on choice models, with many papers also omitting basic outputs/inputs such as model fit, types of draws used, software used, or whether any model selection process had been undertaken (see Appendix 1 for full information and Table A1 for full results).

### Choice Modeling

**Figure 2:**
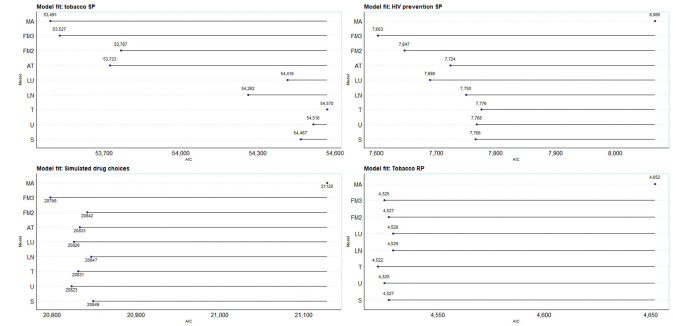
Akaike information criterion (AIC) of models and model averages over 4 datasets. S, standard practice (normal distribution); U, uniform; T, triangular; LN, log normal; LU, log uniform; AT, asymmetric triangular; FM2, Fosgerau and Mabit with second-order polynomials, FM3, Fosgerau and Mabit with third-order polynomials; MA, model averaging. Model averages combine all of the models in each dataset, that is MA3 (S, U, T, LN, LU, AT, FM2, FM3). NB, a conservative approach to AIC for model averaging of counting all parameters from all constituent models, as opposed to only the parameters estimated in the second stage of estimation.

Figure 2 (and Table A2) shows the results from the set of 8 models and model averaging for each of the 4 datasets. For the Akaike information criterion (AIC), lower values denote a better fit, and so values further to the left are superior. In terms of model fit, there are substantial differences across models in 3 of 4 datasets (tobacco SP, HIV prevention, and simulated drug choices) and minimal differences in 1 dataset (tobacco RP). Further, the asymmetric triangular distribution was not estimable on these data; the model collapsed to the triangular distribution. For the tobacco SP data, we extended analyses to the WTP space; Appendix 3 and Table A3 show results for these estimates.

The first analysis is the comparison of model fit (here, the AIC) of standard practice, all normal (S), with alternative distributional assumptions. Standard practice is outperformed in 2 of 4 cases by alternative base models (simulated drug choices and tobacco RP data), in 3 of 4 cases by the extended models (tobacco SP, HIV prevention, and simulated drug choices), in 3 of 4 cases for extended models (tobacco SP, HIV prevention, and simulated drug), and in 1 of 4 cases by the flexible models and model averaging (tobacco SP). In all cases, moving away from standard practice resulted in a better model fit.

An additional analysis reran the standard practice model for tobacco SP omitting each attribute singly as yardsticks against which to pitch gains in fit of alternative distributions. We found losses in LL of 363 units (health harm), 2,320 (flavor), 279 (nicotine), and 1,768 (price). These compared with a difference of 685 units between standard practice and the best fitting model, MA3. Hence, improving the choice of distributional assumption, even in our rather limited form (by assuming all attributes follow the same distribution), improves fit by a comparable amount as a low explanatory attribute.

The second analysis is on the models’ predicted choice probabilities. Table 2 compares the predicted choice probabilities for each model and for model averaging on each of the 4 datasets. There does not appear to be much, if any, impact of either distributional assumptions or model averaging on forecasts from models. Overall, it does not appear that distributional assumptions affect the models’ predicted choice probabilities.

**Table 2:** Estimated Choice Probabilities for Alternatives from 8 Models and Model Averaging on 4 Datasets

Model	Description	Tobacco SP			HIV Prevention		Drug Choice Simulated		Tobacco RP			
		Cigarette	e-Cigarette	Opt-out	Any Product	Opt-out	Branded	Unbranded	Smoker	Nonsmoker	Vaper	Nonvaper
1	Normal	0.50	0.38	0.13	0.68	0.32	0.46	0.54	0.82	0.18	0.38	0.62
2	Uniform	0.48	0.38	0.14	0.71	0.29	0.46	0.54	0.82	0.18	0.38	0.62
3	Triangular	0.50	0.37	0.13	0.68	0.32	0.44	0.56	0.82	0.18	0.38	0.62
4	Log normal	0.48	0.37	0.15	0.66	0.34	0.45	0.55	0.82	0.18	0.38	0.62
5	Log uniform	0.50	0.37	0.13	0.66	0.34	0.46	0.54	0.82	0.18	0.38	0.62
6	Asymmetric triangular	0.51	0.36	0.13	0.67	0.33	0.47	0.53				
7	Fosgereau and Mabit^2^	0.50	0.37	0.13	0.64	0.36	0.46	0.54	0.82	0.18	0.38	0.62
8	Fosgereau and Mabit^3^	0.49	0.38	0.13	0.63	0.37	0.46	0.54	0.82	0.18	0.38	0.62
MA	Model averaging	0.47	0.37	0.16	0.64	0.36	0.46	0.54	0.81	0.19	0.38	0.62

**Figure 3:**
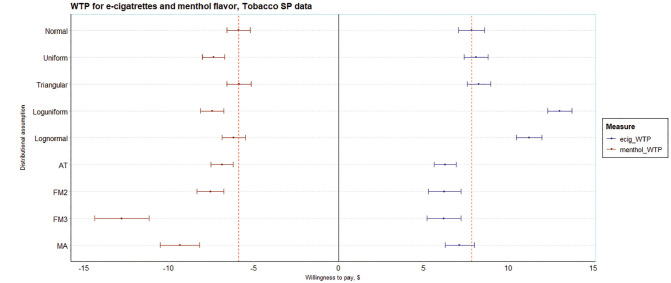
Estimates of willingness to pay (WTP) for e-cigarettes and menthol flavor on the tobacco SP data. Estimates derived from the WTP space model (Eq. 4a).

The third analysis is of WTP, shown in Figure 3, with menthol flavor (reference: tobacco flavor) and e-cigarette (reference: the opt-out in the experiment) preferences taken as examples. These are taken from the WTP space model and are directly estimated without the need for the postestimation calculation of point estimates and standard errors. There is considerable heterogeneity across the mean WTP for both parameters. For menthol, the WTP for the normal distribution is −$5.90 (95% confidence interval [CI]: −$5.21 to −$6.58), which also happens to be the highest. The minimum, from the FM3 model, −$12.78 (95% CI: −$14.38 to −$11.17), is about double that from the normal distribution and statistically significantly different. An FM4 model (−$16.85) was tested and omitted as it had worse Bayesian information criterion but suggests that the estimate for FM3 is not an outlier, perhaps better capturing the tail (implying many individuals have a strong negative preference for menthol; see Figure 3). The model averaging WTP, −$9.35 (95% CI: −$10.51 to −$8.19), is also considerably lower than that of the normal distribution and statistically significantly different. For e-cigarettes, the WTP for the normal distribution, $7.83 (95%CI: $7.07 to $8.59), is neither the highest nor lowest. The WTP from the FM3 is lowest, $6.19 (95% CI: $5.19 to $7.21) and the WTP from the log uniform model is highest, $13.02 (95% CI: $12.32 to $13.73); both are statistically significantly different from the WTP from the normal distribution. The model averaging WTP, $7.13 (95% CI: $6.27 to $7.99), is also lower than that of the normal distribution although not statistically significantly different. As expected, the choice of distributional assumption has important ramifications for the estimates of WTP. WTP was different and for menthol, statistically significantly different for the preferred model (according to AIC) relative to standard practice.

**Figure 4:**
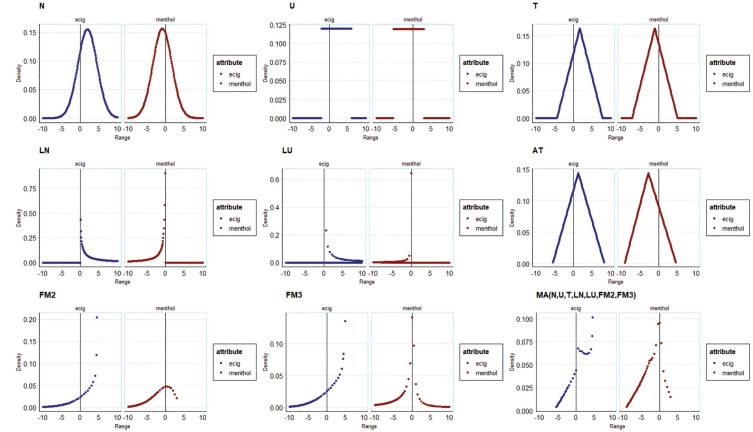
Illustration of preference distributions from tobacco SP models for e-cigarettes and for menthol, across different distributional assumptions and model averages.

A fourth analysis of preference distributions is presented in Figure 4. This shows the probability densities for e-cigarette (reference: the opt-out in the experiment) and menthol (reference: tobacco) preferences. The shapes of the distributions for the base models resemble the impositions made on them. The shapes of the preference distributions in the extended models differ and embody the unidirectionality imposed by their specifications. In the flexible models, the shapes of the distributions are substantially different and allow for asymmetry in the preference distributions and multimodality in the FM models. For model averaging, the shape of the distribution reflects the model specification in that they are weighted averages of the probability densities of, that is, distributions imposed by, the constituent models. The shapes of the distributions are similar for some models (e.g., normal versus triangular) and very different for others (e.g., normal versus FM3). For preferred models, the shapes of the distributions are very different from those recovered from applying standard practice.

**Figure 5:**
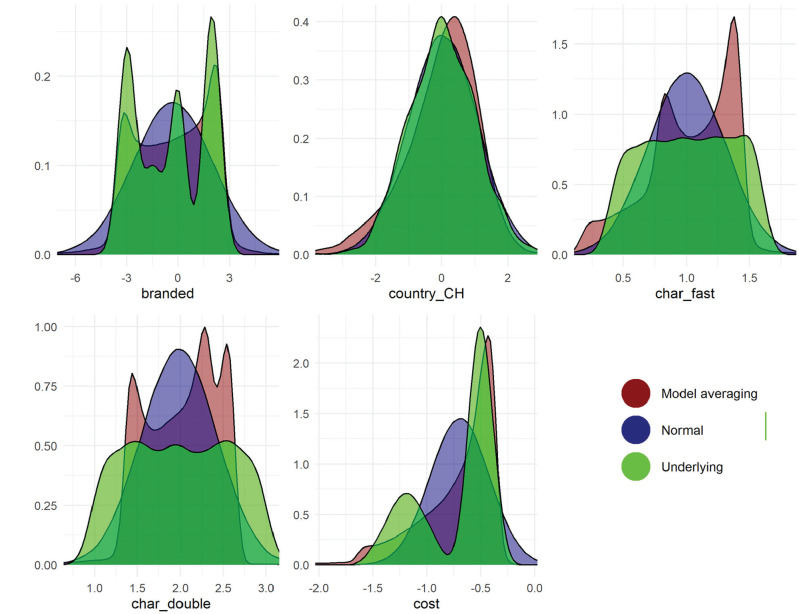
Distributions of preferences for different attributes in the simulated drug choice data. The underlying distribution is in green and varies across attributes. The distribution from model averaging is given in red and the normal distribution is in blue. This figure uses the MA3(S,U,T,LN,LU,AT,FM2,FM3) model, which draws from the full set of models.

Finally, the recoverability of underlying preference distributions in the simulated dataset are illustrated in Figure 5. This figure shows 1) the underlying distribution, 2) the fitted normal distribution, and 3) the distribution of the best-fitting model (MA). MA, given its flexibility, captures multimodality in preferences for “branded.” This was not the case for the normal distributions. Normal distributions fitted similarly to MA when the underlying preferences were themselves normal (e.g., country_CH). By definition, as visible in the figure, normal distributions do not recover multimodality in preference distributions. This demonstrates the effectiveness of model averaging over more complex distributions in capturing the underlying distributions.

## Discussion

In this article, we considered random preference heterogeneity in discrete choice models in health-based choice modeling. A scoping review of the literature established current practices in modeling (and reporting of modelling). There is no defined reporting standard for choice modeling. The most commonly used model in health is the mixed logit. Standard practice in health is to use normal mixing distributions for all parameters in models. We show that there are better alternatives that consistently fit the data better and have significantly different model outputs, implying that standard practice may give biased outputs.

With 4 datasets, 8 specifications of mixing distributions were compared, including standard practice, in multinomial logit and logit-based choice models. These ranged from simple assumptions to flexible approaches (the latter introduced here in health). We also used model averaging as a simple method to reduce analyst bias.

Alternative distributional assumptions offered some gains in model fit to standard practice in all 4 settings. Flexible approaches offered the largest gains in fit among individual models; model averaging improved model fit further in 1 of 4 cases.

Alternative distributional assumptions did not affect in-sample predicted choice probabilities across the datasets studied. Model averaging likewise did not affect choice probabilities (likely because it is drawing from these models). Note, however, that model averaging has been shown to improve out-of-sample forecasting in all datasets tested^20,40^; thus, we opted not to repeat this test here.

Alternative distributional assumptions yielded a wide range of WTP estimates, many of which were statistically significantly different to those derived using normal distributions.

Alternative distributional assumptions yielded a wide range of preference distributions, which allowed for both asymmetry and multimodality, which the normal distribution does not; they are further able to avoid the fact that normal distributions have long tails, which implies extreme preferences (an assumption that may not be warranted).

Aside from predicted choice probabilities, these results raise serious concerns for standard practice in health-based choice modeling. Normal distributions were inferior specifications in terms of model fit in all cases. Not only did the preference distributions of alternative assumptions depart markedly from normal distributions, our simulations indicated that alternatives were far better able to recover the true underlying distributions in the data. The differences observed in WTP in alternative distributions suggests that those derived from models using normal distributions are likely to be biased to varying degrees. This brings into question the robustness of findings of standard practice.

Our scoping review revealed the dominant use of prepackaged software among those reported. This may be limiting if routines are not available for the full range of functional forms, and default settings may inadvertently dictate the choice of distribution. Free software, with code, is now available for researchers to use the alternatives studied here and further specifications that are not (e.g., higher-order polynomials).

The strengths of our study include the scoping review to document current practices and inform standard practice. We used 4 datasets comprising stated preferences, revealed preferences, and simulated data. We used the common multinomial logit model for health choices but also extended our methods to include logistic regression with correlated errors. We introduced new flexible mixing distributions to health, as well as model averaging, which we have shown to offer substantial gains. The use of simulated data means that not only are we able to share code for estimating the models, but researchers can download the data and replicate these results.

Our study is subject to a set of limitations. First, we did not include models with latent classes in our modeling exercises. This is partly due to the primacy of mixed logit modeling in the literature and partly due to keeping the research questions focused. As per our introduction, there are many other model structures that analysts could use: including allowing correlation across mixing distributions,^10^ latent classes,^11^ mixed latent class models,^12^ logit mixed logit models,^13^ and nonparametric approaches.^14^ Some of these, for example, correlated mixing distributions and mixed latent classes, are easily implemented in software packages. We also did not use deterministic heterogeneity in our analyses, which is standard practice in choice modeling. This is in part to keep the exposition simple and the number of estimated models manageable and in part to reflect current practices in health, which infrequently use deterministic heterogeneity. We further recognize that model averaging will require additional correction for standard errors given that there are 2 stages in estimation. We leave this issue for future research. We further note that that model averaging is limited to the space of its constituent models. We were limited to using as few as 100 or 500 draws in estimation. This was a consequence of limited computing power and, in the case of the tobacco RP data, stability in estimates. It is well known that more draws yield more reliable results.^41,42^ We used only parametric approaches (and semi-nonparametric in the case of FM2 and FM3). There are nonparametric approaches that are also available and should be investigated.^22^ We were unable to estimate the AT model on the revealed preference data, which has a fairly large sample size of more than 2,000 observations. Other revealed preference datasets with fewer observations may find difficulties in estimating the more complex specifications. Model averaging did not aid this issue in this setting. Finally, specifications in which all parameters had the same distributions applied to them were considered; distributions can of course be specified on a parameter-by-parameter basis, implying that further gains in fit may be possible.

We refrain from providing specific guidance in this article for several reasons. First, it is not quite clear what guidance could be given at all. For example, consider the case of advice on when to use log-transformed distributions. A lot of choice modelers would use these for a cost attribute based on economic theory. We could extend this to health for, say, health harms (since it could be argued that preferences for these should be strictly negative). Yet, 2 issues arise. First, that some individuals may prefer more harmful products. For example, some hardened smokers may consider reduced-harm products to be a sign of weakness (as per Thirlway’s^43^ narratives) and therefore less appealing. Second, we may wish to first use a distribution that allows preferences to cross zero. Even contrary to expectations, it still may be that a reasonable proportion of preferences cross zero and that may be a signal of data quality issues (e.g., omitted variable bias). Second, we are concerned that guidance and suggestions are treated as direct instructions and may become norms. This may not be good for future research and in essence is the practice that we are trying to challenge here (i.e., using “all normals” is the current default practice). Third, that our results are varied and hence there is little opportunity to give guidance beyond “be sure to test the shape of the distribution that you use,” which we hope is self-evident from our findings. In some cases, it should be noted that the distributions tested here are not helpful, as they may not have the required flexibility to accurately capture preferences (e.g., if we expect both positive and negative preferences for a given attribute, log normals are clearly inadequate). Conversely, distributions may have too much flexibility (e.g., infinite support) and may be unrealistic. Case-specific domain knowledge is required.

If the goal of the research is forecasting behaviors, our results suggest that there is no imperative to move away from normal mixing distributions. However, this is seldom the case, and there are many settings in which preferences, preference distributions, and WTP measures are required; for example, using WTP for cost–benefit analyses or valuation of nonmarket goods. In these settings, the use of normal mixing distributions is highly questionable and likely yields inaccurate model outputs. Alternative approaches can outperform standard practice, better approximate underlying preference distributions, and yield more reliable measures of WTP.

Normal distributions are used for mixing distributions in choice models in almost all cases in health. This evidence suggests that alternative approaches can better capture preference distributions and produce more reliable estimates from choice models.

## Supplemental Material

sj-docx-1-mdm-10.1177_0272989X251357879 – Supplemental material for A Break from the Norm? Parametric Representations of Preference Heterogeneity for Discrete Choice Models in Health Supplemental material, sj-docx-1-mdm-10.1177_0272989X251357879 for A Break from the Norm? Parametric Representations of Preference Heterogeneity for Discrete Choice Models in Health by John Buckell, Alice Wreford, Matthew Quaife and Thomas O. Hancock in Medical Decision Making
