# Supplementary material for: A Break from the Norm? Parametric Representations of Preference Heterogeneity for Discrete Choice Models in Health
Source: Med Decis Making. 2025 Sep 5;45(8):987–1001. doi: 10.1177/0272989X251357879 (PMC12511644; doi:10.1177/0272989X251357879)
Supplement: sj-docx-1-mdm-10.1177_0272989X251357879 – Supplemental material for A Break from the Norm? Parametric Representations of Preference Heterogeneity for Discrete Choice Models in Health [file sj-docx-1-mdm-10.1177_0272989X251357879.docx]

Appendices

Appendix 1: scoping review

We conducted a scoping review of choice modelling in health in 2022 to get a view of current practices in the field (Appendix 1 provides the full details of the scoping review). We also searched the year 2017 to observe any notable changes in practices over the period. A preliminary set of search terms were found in Clark et al. (2014) and Vass et al., (2022). To ensure search terms aligned with recent developments in the choice modelling literature, search terms were iterated between research team members and an academic librarian. Further, search results were compared to known papers [for inclusion]. For example, the absence of “choice modelling” and “choice behaviour” (with truncation relevant to each database) overlooked a number of relevant papers. These terms were not included in previous reviews. We searched EconLIT (limited to Health and social care), PubMED and the Journal of Choice Modelling (through Scopus). The complete search strategy is presented in the supplementary information.

Given the extensive number of papers including choice modelling work, nesting strategies were employed to limit search results. Papers which mentioned models, known for assessing preference heterogeneity, and parametric distributions were sought. Noting that modelling approaches were seldom reported in the abstract, title or keywords, and comprehensive full text searching relied upon html upload of the paper’s pdf to the search databases, we note that this strategy is not exhaustive. To mitigate the effect of this limitation, restrictions on search terms “latent” and “logit” were eased; papers that mention them, without the mention of a distributional assumption, were retrieved. A process of reference mining was employed to further capture known papers not retrieved in the strategy.

Papers were included if they reported use of a discrete choice experiment (or relevant synonym) and considered preference heterogeneity in their modelling approaches. Papers were included if they were relevant to health and/or social care, including health behaviours. Papers were excluded if they were investigating disease control approaches to the COVID-19 pandemic through transport or policy not relevant to vaccination programmes. Consumer behaviour studies were excluded unless specifically comparing health outcomes. English language papers only were considered.

Search results were uploaded to online collaborative working platform Rayyan (<https://www.rayyan.ai/>). Researcher AW conducted an initial title and abstract screen, with 20% overlap (10% overlap JB, 10% overlap TH). Conflicts were resolved in group conversation. A conservative approach was taken, such that if the paper did not mention modelling technique in the abstract of title, the paper would move forward to the second full text screen. Remaining papers were divided between researchers (AW, JB, TH, MQ) for full text review, with 10% overlap. Queries or conflicts were resolved through group conversation.

A data extraction sheet was prepared in Microsoft Excel with headings: ‘Model type’, ‘Reporting of diagnostics’, ‘MMNL features’, ‘LC features’, ‘Model selection testing’, ‘Number of models’, ‘Software, package and version used’ and ‘Notes’. Researchers extracted diagnostics and model feature data on the ‘best’ model within each publication. The hierarchical criterion for choosing the best model was as follows:

1. The model presented in the paper
2. Akaike/Bayesian information criterion (AIC/BIC)
3. Adjusted Rho squared ($\rho^{2}$)
4. Log likelihood (LL)
5. Most complex model

In instances where equivalent models were applied to multiple population groups or subgroup analysis, one model was counted. The researchers populated the excel sheet, noting “not reported” in instances where the paper does not mention the relevant information. Queries or conflicts were resolved in a group conversation.

Search strategy

PubMED

(

(“Conjoint”[tiab] OR “Part-worth utilit*”[tiab] OR “Functional measuremen*”[tiab] OR “Paired compariso*”[tiab] OR “Pairwise choic*”[tiab] OR “Discrete choic*”[tiab] OR “Stated preferenc*”[tiab] OR “Revealed preferenc*”[tiab] OR “Preference heterogenei*”[tiab] OR “choice mode*”[tiab] OR “Choice behav*”[tiab] OR “hybrid choic*”[tiab])

AND

(

(“Finite mixtur*”[tw] OR “Mixture mod*”[tw] OR “logi*” [tw] OR “Latent” [tw] OR “Probi*”[tw] OR “Baye*”[tw])

OR

(

(“Random param*”[tw] OR “Random slop*”[tw] OR “Random intercep*”[tw] OR “Random coeff*”[tw] OR “logi*”[tw] OR “Mixing distributio*”[tw] OR “Error componen*”[tw])

AND

(“Norma*”[tw] OR “Lognorma*”[tw] OR “Uniform”[tw] OR “Loguniform”[tw] OR “Triangular”[tw] OR “Asymmetric triangular”[tw] OR “Johnson SB” OR “Johnsons SB”[tw] OR “Fosgera*”[tw])

)))

EconLit

(TX(Healt* OR socia*)
AND
( (

(TX (“conjoint” OR Part#worthutilit* OR Functional measuremen* OR Functional measuremen* OR Paired compariso* OR Pairwise choic* OR Discrete choic* OR Stated preferenc* OR Revealed preferenc*OR Preference hetrogenei*OR choice mode* OR Choice behav* OR hybrid choic*))

AND

(

TX (Finite mixtur* OR Mixture mod* OR logi* OR “Latent” OR Probi* OR Baye* ))

OR

(

TX(Random param* OR Random slop* OR Random intercep* OR Random coeff* OR logi* OR Mixing distributio* OR Error componen*))

AND

(TX (Norma* OR Lognorma* OR “Uniform” OR “Loguniform”  OR “Triangular” OR “Asymmetric triangular” OR Johnso* SB OR Fosgera*))

))))

Journal of choice modelling (retrieved through SCOPUS)

(ALL(“Healt*”) OR ALL(“socia*”))

AND

((TITLE-ABS-KEY({conjoint}zz) OR TITLE-ABS-KEY(“Part-worth utilit*”) OR TITLE-ABS-KEY(“Functional measuremen*”) OR TITLE-ABS-KEY(“Paired compaiso*”) OR TITLE-ABS-KEY(“Pairwise choic*”) OR TITLE-ABS-KEY(“Discrete choic*”) OR TITLE-ABS-KEY(“Stated preferenc*”) OR TITLE-ABS-KEY(“Revealed preferenc*”) OR TITLE-ABS-KEY(“Preference heterogenei*”) OR TITLE-ABS-KEY(“choice mode*”) OR TITLE-ABS-KEY(“choice behave*”) OR TITLE-ABS-KEY(“hybrid choic*”))

AND

(

((ALL({Latent}) OR ALL(“Finite mixtur*”) OR ALL(“Mixture mod*”) OR ALL(“logi*”) OR ALL(“Probi*”) OR ALL(“Baye*”))

OR

(

(ALL(“Random param*”) OR ALL(“Random slop*”) OR ALL(“Random intercep*”) OR ALL(“Random coeff*”) OR ALL(“logi*”) OR ALL(“Mixing distributio*”) OR ALL(“Error componen*”))

AND

(ALL(“Norma*”)  OR ALL(“Lognorma*”) OR ALL({uniform}) OR ALL({loguniform}) OR ALL({triangular}) OR ALL({asymmetric triangular}) OR ALL ({Johnsons SB}) OR ALL({Johnson SB}) OR ALL(“Fosgera*”))))))

Results

|  | | **2017** | | | | | **2022** | | | | | **Total**  **n=207** |
| --- | --- | --- | --- | --- | --- | --- | --- | --- | --- | --- | --- | --- |
|  |  | **Modelling approach** | | | | **All model types n=60** | **Modelling approach** | | | | **All model types n= 147** |  |
|  |  | **LC n=16** (27%) | **MMNL (SML)**  **n=35**  (58%) | **MMNL (HB) n=8**  (13%) | **LC MMNL (SML) n=1** (2%) |  | **LC n=30**  (20%) | **MMNL (SML)**  **n= 105**  (71%) | **MMNL (HB) n=8**  (5%) | **LC MMNL (SML) n=4**  (3%) |  |  |
| **Papers reporting No. of random parameters** | | n/a | 30 (86%) | 5 (63%) | 1  (100%) | **36 (82%^[[1]](#footnote-1)^)** | n/a | 94 (90%) | 7 (88%) | 3 (75%) | **104**  **(89%^1^)** | **140**  **(87%^1^)** |
| **Total No. of random parameters** | | n/a | 365 | 47 | 8 | **420** | n/a | 943 | 41 | 45 | **1,029** | **1,449** |
| **Distributions of RP** | Normal | - | 191 (52%) | 35  (74%) | - | **226**  **(54%)** | - | 442 (47%) | 24  (59%) | 44  (98%) | **510**  **(59%)** | **736**  **(51%)** |
|  | Uniform | - | - | - | - | **-** | - | - | - | - | **-** | **-** |
|  | Triangular | - | - | - | - | **-** | - | - | - | - | **-** | **-** |
|  | Log normal | - | 3 (1%) | 1  (2%) | - | **4**  **(1%)** | - | 5 (1%) | - | 1  (2%) | **6**  **(1%)** | **10**  **(1%)** |
|  | Log uniform | - | - | - | - | **-** | - | - | - | - | **-** | **-** |
|  | Fosgreau & Mabit | - | - | - | - | **-** | - | - | - | - | **-** | **-** |
|  | Unspecified | - | 171 (47%) | 11 (23%) | 8 (100%) | **190**  **(45%)** | - | 496  (53%) | 17  (41%) | - | **513**  **(50%)** | **703**  **(49%)** |

Table A1: Results from scoping review of random heterogeneity in health choice modelling in the years 2017 and 2022. LC – latent class; MMNL (SML) – mixed multinomial logit model, simulated maximum likelihood; MMNL (HB) – mixed multinomial logit model, hierarchical Bayes; LC MMNL (SML) – latent class, mixed multinomial logit model, simulated maximum likelihood.

Table A1 shows the results for types of distributions used in mixed logit models retrieved in our search. In 2017, 98% (226/230) of all distributions were normal with 2% (4/230) being lognormal. In 2022, 99% (736/746) of all distributions were normal with 1% (10/746) being lognormal. Based on this, we define a “standard practice” in health to be using normal distributions for all parameters. Notably, almost half of papers did not report the distributional assumptions used in their model. This was part of a worrying theme of not reporting essential information on choice models, with many papers also omitting basic outputs/inputs such as model fit, types of draws used, software used or whether any model selection process had been undertaken (see below for full information; and Table A2 for full results).

|  | | **2017** | | | | | **2022** | | | | | **Total**  **n=207** |
| --- | --- | --- | --- | --- | --- | --- | --- | --- | --- | --- | --- | --- |
|  |  | **Modelling approach** | | | | **All model types n=60** | **Modelling approach** | | | | **All model types n= 147** |  |
|  |  | **LC n=16** (27%) | **MMNL (SML)**  **n=35**  (58%) | **MMMNL (HB) n=8**  (13%) | **LC MMNL (SML) n=1** (2%) |  | **LC n=30**  (20%) | **MMNL (SML)**  **n= 105**  (71%) | **MMMNL (HB) n=8**  (5%) | **LC MMNL (SML) n=4**  (3%) |  |  |
| **Journal Classification** | Economics | 1  (6%) | 1  (3%) | - | - | **2 (3%)** | - | 2  (2%) | - | - | **2**  **(1%)** | **4**  **(2%)** |
|  | Health Economics | 3  (19%) | 12  (34%) | 3  (38%) | 1  (100%) | **19**  **(32%)** | 11  (37%) | 34  (32%) | 3  (38%) | 2  (50%) | **50**  **(33%)** | **69**  **(33%)** |
|  | Non Economics/ Health economics | 12  (75%) | 22  (63%) | 5  (62%) | - | **39**  **(65%)** | 19  (63%) | 69  (66%) | 5  (62%) | 2  (50%) | **95**  **(65%)** | **134**  **(65%)** |
| **Papers reporting N^o^  of RP** | | n/a | 30 (86%) | 5 (63%) | 1  (100%) | **36 (82%^[[2]](#footnote-2)^)** | n/a | 94 (90%) | 7 (88%) | 3 (75%) | **104**  **(89%^1^)** | **140**  **(87%^1^)** |
| **Total N^o^ of RP** | | n/a | 365 | 47 | 8 | **420** | n/a | 943 | 41 | 45 | **1,029** | **1,449** |
| **Distributions of RP** | Normal | - | 191 (52%) | 35  (74%) | - | **226**  **(54%)** | - | 442 (47%) | 24  (59%) | 44  (98%) | **510**  **(59%)** | **736**  **(51%)** |
|  | Uniform | - | - | - | - | **-** | - | - | - | - | **-** | **-** |
|  | Triangular | - | - | - | - | **-** | - | - | - | - | **-** | **-** |
|  | Log normal | - | 3 (1%) | 1  (2%) | - | **4**  **(1%)** | - | 5 (1%) | - | 1  (2%) | **6**  **(1%)** | **10**  **(1%)** |
|  | Log uniform | - | - | - | - | **-** | - | - | - | - | **-** | **-** |
|  | Fosgreau & Mabit | - | - | - | - | **-** | - | - | - | - | **-** | **-** |
|  | Unspecified | - | 171 (47%) | 11 (23%) | 8 (100%) | **190**  **(45%)** | - | 496  (53%) | 17  (41%) | - | **513**  **(50%)** | **703**  **(49%)** |
| **Type of draws; range of number of draws** | Gibbs | - | - | 1 (13%); 100,000 | - | **1 (2%); 100,000** | - | - | - | - | **-** | **1 (<0.5%); 100,000** |
|  | Halton | - | 17 (49%); 500 -5,000 | - | - | **17 (28%) 500 – 5,000** | 1 (3%); 1,000 | 26 (25%); 100 – 140,000 | 1 (13%); 500 | 1 (25%); 1,000 | **29 (20%); 100 - 140,000** | **46 (22%);**  **100 - 140,000** |
|  | Scrambled Halton | - | - | - | - | **-** | - | 1 (1%); 8,000 | - | - | **1 (1%); 8,000** | **1 (<0.5%); 8,000** |
|  | Sobol | - | - | - | - | **-** | - | - | - | 1 (25%); 5,000 | **1 (1%); 5,000** | **1 (<0.5%); 5,000** |
|  | Pseudo random | - | - | - | - | **-** | - | 2 (2%); 2,000 -5,000 | - | - | **2 (1%); 2,000, 5,000** | **2 (1%); 2,000- 5,000** |
|  | MCMC | - | - | 3 (38%); 3,000-30,000 | - | **3 (5%); 3,000 – 30,000** | - | - | - | - | **-** | **3 (1%); 3,000 – 30,000** |
|  | MHLS | - | - | - | - | **-** | - | 2 (2%); 500 - 3,000 | - | - | **2 (1%); 500 - 3,000** | **2 (1%); 500 - 3,000** |
|  | Unreported | 16 (100%) | 18 (51%) | 4 (50%) | 1 (100%) | **39  (65%)** | 29  (97%) | 74 (70%) | 7  (88%) | 2  (50%) | **112 (76%)** | **151**  **(73%)** |
| **Measure of model fit** | LL | 5 (31%) | 13  (37%) | 1  (13%) | - | **19**  **(32%)** | 11  (37%) | 53  (50%) | 1  (13%) | 3  (75%) | **68**  **(46%)** | **87**  **(42%)** |
|  | BIC/AIC | 1 (6%) | 1  (3%) | - | - | **2**  **(3%)** | 2  (7%) | 4  (4%) | - | - | **6**  **(4%)** | **8**  **(4%)** |
|  | “Did not converge” reported | - | - | - | - | **-** | - | 1  (1%) | - | - | **1**  **(1%)** | **1 (>0.5%)** |
|  | Unreported | 10  (63%) | 21  (60%) | 7  (88%) | 1  (100%) | **39**  **(65%)** | 17  (57%) | 47 (45%) | 7  (88%) | 1  (25%) | **72**  **(49%)** | **111**  **(54%)** |
| **Report model selection testing reported** | | 12 (75%) | 12  (34%) | - | - | **24**  **(40%)** | 22  (73%) | 39  (38%) | 1  (13%) | 3  (75%) | **65**  **(44%)** | **89**  **(43%)** |
| **Software used** | BUGS | - | - | - | - | **-** | 1 (3%) | - | - | - | **1**  **(1%)** | **1**  **(<0.5%)** |
|  | Gauss | 1 (6%) | - | - | - | **1 (2%)** | - |  | - | - | **-** | **1**  **(<0.5%)** |
|  | JAGS | - | - | 1 (13%) | - | **1 (2%)** | - | - | - | - | **-** | **1**  **(<0.5%)** |
|  | JMP | - | - | - | - | **-** | - | - | 2 (25%) | - | **2**  **(1%)** | **2**  **(1%)** |
|  | Laten Gold | 4  (25%) | - | - | - | **4 (7%)** | 1 (3%) | - | - | - | **1**  **(1%)** | **5**  **(2%)** |
|  | MATLAB | - | - | 1 (13%) | - | **1 (2%)** | - | - | - | - | **-** | **1**  **(<0.5%)** |
|  | MaxDiff | 1 (6%) | - | - | - | **1 (2%)** | - | - | - | - | **-** | **1 (<0.5%)** |
|  | Mplus | 1 (6%) | - | - | - | **1 (2%)** | - | - | - | - | **-** | **1**  **(<0.5%)** |
|  | Ngene | - | - | - | - | **-** | - | 1 (1%) | - | - | **1**  **(1%)** | **1 (<0.5%)** |
|  | Nlogit | 1 (6%) | 15  (43%) | - | - | **16 (27%)** | 4 (13%) | 7 (7%) | 1 (13%) |  | **12**  **(8%)** | **28**  **(14%)** |
|  | OpenBUGS | - | - | 1 (13%) | - | **1 (2%)** | - | - | - | - | **-** | **1**  **(<0.5%)** |
|  | Python | - | - | - | - | **-** | - | 2 (2%) | - | - | **2**  **(1%)** | **2**  **(1%)** |
|  | SAS | - | - | - | - | **-** | - | 1 (1%) | - | - | **1**  **(1%)** | **1 (<0.5%)** |
|  | Sawtooth | 1 (6%) | - | 1 (13%) | - | **2 (3%)** | 5  (17%) | - | 2  (25%) | 1 (25%) | **8**  **(5%)** | **10 (5%)** |
|  | SPSS | 2  (13%) | - | - | - | **2 (3%)** | 1 (3%) | 1 (1%) | - | - | **2**  **(1%)** | **4**  **(2%)** |
|  | Stata | - | 9 (26%) | 2 (25%) | - | **11**  **(18%)** | 6 (20%) | 54 (51%) | 2  (25%) | - | **62**  **(42%)** | **73**  **(35%)** |
|  | R | - | - | - | - | **-** | 2 (7%) | 10 (10%) | - | 1  (25%) | **13**  **(9%)** | **13**  **(6%)** |
|  | WinBUGS | - | - | 1 (13%) | - | **1 (2%)** | - | - | - | - | **-** | **1**  **(<0.5%)** |
|  | Unreported | 5  (31%) | 11 (31%) | 1 (13%) | 1 (100%) | **18 (30%)** | 10  (33%) | 29 (27%) | 1  (13%) | 2  (50%) | **42**  **(29%)** | **60**  **(29%)** |

Table A2: full results from scoping review, years 2017 and 2022

Searches yielded a total of 1,013 results, of which 63 were duplicates. Therefore, 950 abstracts were screened. Of the screened abstracts 654 were excluded. The remaining 296 went forward to full text screen. 207 papers were elected for inclusion.

A summary of the process of review and selection is given in PRISMA flow diagram in Figure 1.

**
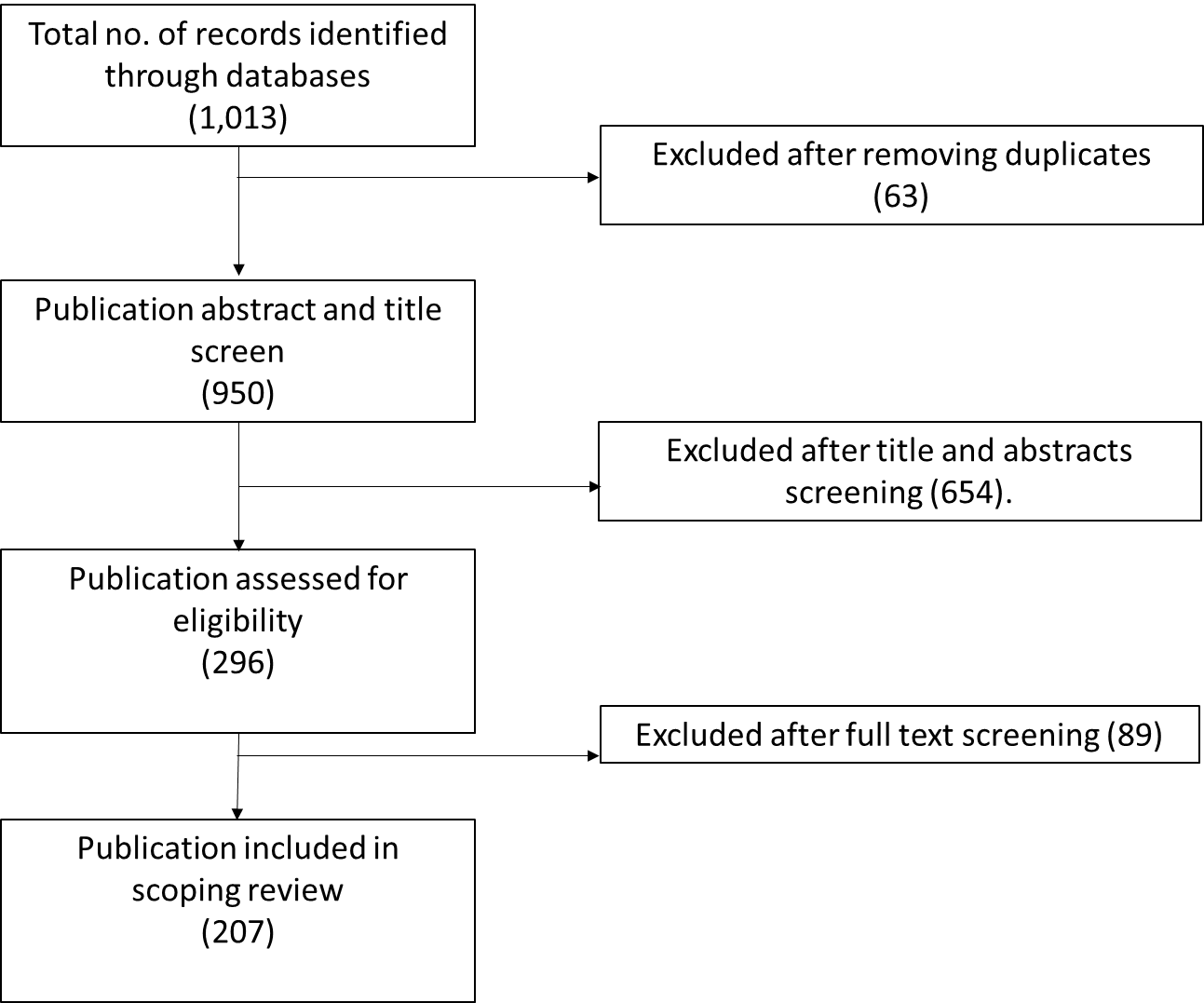
**

Figure 1: PRISMA chart from results of scoping review

Of the 207 (2017: 60; 2022: 147) included studies, latent class [LC, n=46 (2017: 16, 27%; 2022: 30, 20%)], mixed MNL (simulated maximum likelihood) [MMNL (SML), n=140 (2017: 35, 58%; 2022: 105, 71%)], mixed MNL (hierarchical Bayes) [MMNL (HB), n=16 (2017: 8, 13%; 2022: 8, 5%)] and latent class mixed MNL (simulated maximum likelihood) [LC MMNL (SML), n=5 (2017: 1, 2%; 2022: 4, 3%)] models were presented. Each modelling technique will be considered in turn, as summarised in Table xxx.

LC was used in 46 publications (2017: 16, 27%; 2022: 30, 20%), with an almost two-fold increase between years 2017 and 2022. In all but one instance draws were unreported. A single publication from 2022 reported use of Halton draws (1,000 draws). Measure of model fit was predominately unreported (2017: 10, 63%: 2022: 17, 57%). Loglikelihood (LL) was reported in 16 studies (2017: 5, 31%; 2022: 11, 37%), and Bayesian Information Criterion (BIC) was reported in place of LL in 6-7% of publications within each year. Model selection testing was reported in 73-75% of publications; with multiple models presented (typically with varied number of classes).

Of the papers presenting MMNL (SML) [n=140 (2017: 35, 58%; 2022: 105, 71%)], sixteen (2017: 5, 14%; 2022: 11, 10%) did not report their parametric assumption. Between years 2017 and 2022 the proportion of papers reporting such detail increased from 86% to 90%. Of the papers reporting parametric detail, a total of 1,308 random parameters (2017: 365; 2022: 943) were reported. 663 (2017:191, 52%; 2022: 442, 47%) were normally distributed; 8 (2017: 3, 1%; 2022: 5, 1%) were lognormal and 667 (2017: 171, 47%; 2022: 496, 53%) were unspecified. The type of draws used was unreported in 92 (2017: 18, 51%; 2022 75, 70%) publications. Across both years Halton draws (100 – 140,000 draws) dominated, reported in 43 (2017: 17, 49%; 2022: 26, 25%) publications. MCMC (n=3, 3,000- 30,000 draws) and Gibbs (n=1, 100,00 draws) were also reported in 2017. Pseudo random (n=2, 2000 and 5000 draws), MHLS (n=1, 500 draws) and Scrambled Halton (n=1, 8000 draws) draws were present in 2022. Assessment of model fit was reported via LL [n=66 (2017: 13, 37%; 2022: 53, 50%)] and BIC [n=5 (2017: 1, 3%; 2022: 4, 4%)], with 68 (2027: 21, 60%; 2022: 47, 45%) not reporting any measure of model fit. Model selection testing was reported in a single publication (in 2022).

MMNL (HB) [n=16 (2017: 8, 13%; 2022: 8, 5%)] models made use of 88 (2017: 47; 2022: 41) reported random parameters: 59 (2017: 35, 74%; 2022: 24, 59%) were normally distributed; one lognormal (2017); and, the remaining unspecified [n=28 (2017: 11, 23%; 17, 41%)]. LL, was the only presented measure of model fit, shown in only a single study for each year.

LC MMNL (SML) was presented as the best fit model in five papers (2017: 1, 2%; 2022: 4, 3%). Random parameters were reported in 4 (2017: 1; 2022: 3) of the papers. Of the 53 reported random parameters: 44 (2022, 98%) were normally distributed; one was log normal (2022, 2%); and, 8 were unspecified (2017: 100%). Sobol (5,000 draws) and Halton (1,000 draws) draws were reported across two papers in 2022, with all others unreported. Three LC MMNL (SML) publications (in 2022) reported LL measure of model fit, and model selection testing.

Appendix 2: model parameters and log-likelihoods from all four datasets

|  |  |  | Tobacco SP | | |  | HIV prevention SP | | |  | Drug choice simulated | |  |  | Tobacco RP | |  |
| --- | --- | --- | --- | --- | --- | --- | --- | --- | --- | --- | --- | --- | --- | --- | --- | --- | --- |
|  |  |  |  |  |  |  |  |  |  |  |  |  |  |  |  |  |  |
| Model | Group |  | Pars | LL | AIC |  | Pars | LL | AIC |  | Pars | LL | AIC |  | Pars | LL | AIC |
|  |  |  |  |  |  |  |  |  |  |  |  |  |  |  |  |  |  |
| 1. Normal (N) | Base models |  | 22 | -27212 | 54467 |  | 32 | -3851 | 7766 |  | 21 | -10404 | 20849 |  | 10 | -2253 | 4527 |
| 2. Uniform (U) | Base models |  | 22 | -27236 | 54516 |  | 32 | -3852 | 7768 |  | 21 | -10391 | 20823 |  | 10 | -2253 | 4525 |
| 3. Triangular (T) | Base models |  | 22 | -27263 | 54570 |  | 32 | -3856 | 7776 |  | 21 | -10394 | 20831 |  | 10 | -2251 | 4522 |
| 4. Lognormal (LN) | Extended base models |  | 22 | -27109 | 54262 |  | 32 | -3843 | 7750 |  | 21 | -10403 | 20847 |  | 9 | -2256 | 4529 |
| 5. Log uniform (LU) | Extended base models |  | 22 | -27186 | 54416 |  | 30 | -3815 | 7690 |  | 21 | -10392 | 20826 |  | 9 | -2255 | 4529 |
| 6. Asymmetric triangular (AT) | Flexible models |  | 25 | -26837 | 53723 |  | 34 | -3828 | 7724 |  | 23 | -10394 | 20833 |  |  |  |  |
| 7. Fosgereau & Mabit ^2 (FM2) | Flexible models |  | 33 | -26851 | 53767 |  | 48 | -3775 | 7647 |  | 32 | -10389 | 20842 |  | 11 | -2253 | 4527 |
| 8. Fosgereau & Mabit ^3 (FM3) | Flexible models |  | 44 | -26719 | 53527 |  | 64 | -3737 | 7603 |  | 43 | -10356 | 20798 |  | 12 | -2250 | 4525 |
| MA1: Base models  (MA(S,U,T)) | Model averaging |  | 68 | -26983 | 54102 |  | 98 | -3817 | 7831 |  | 65 | -10384 | 20898 |  | 32 | -2251 | 4565 |
| MA2: Base models + extended  (MA(S,U,T,LN,LU)) | Model averaging |  | 114 | -26920 | 54068 |  | 162 | -3798 | 7920 |  | 109 | -10382 | 20982 |  | 52 | -2251 | 4605 |
| MA3: Base models + extended + flexible  (MA(S,U,T,LN,LU,AT,FM2,FM3)) | Model averaging |  | 219 | -26527 | 53491 |  | 311 | -3722 | 8066 |  | 210 | -10354 | 21128 |  | 77 | -2249 | 4652 |
|  |  |  |  |  |  |  |  |  |  |  |  |  |  |  |  |  |  |

Table A3: Model estimates from 11 models on four datasets. Pars – number of estimated parameters; LL – log-likelihood; AIC – Akaike Information Criterion. All models used 500 Modified Latin Hypercube Sampling (MLHS) draws except for tobacco RP which used 100.

Appendix 3: Tobacco SP dataset results from willingness-to-pay space models

To enable direct computation of wiliness-to-pay (WTP), and WTP distributions, we estimate models in the WTP space (Train and Weeks, 2005) for the tobacco SP dataset. Thus, parameter estimates directly measure marginal rates of substitution between the attribute at hand and price, and can be interpreted as the WTP value of each parameter.

$V_{nti}=\beta_{p} . (-{Price}_{nti}+ {ASC}_{cig}.{Cig}_{nti}+{ASC}_{ecig}.{Ecig}_{nti}++\beta_{N}.{Nicotine}_{nti}+\beta_{f}.{Flavor}_{nti}+\beta_{h}.{Heath Harm}_{nti})$

Where mixing distributions are applied in the same way as in the main paper.

| Model | Group | Description | Shorthand | Pars | LL | AIC |  | Share: s | Share: u | Share: t | Share: ln | Share: lu | Share: at | Share: fm2 | Share: fm3 |
| --- | --- | --- | --- | --- | --- | --- | --- | --- | --- | --- | --- | --- | --- | --- | --- |
| 1 | Standard practice | All normal | N | 22 | -27489.18 | 55022 |  |  |  |  |  |  |  |  |  |
| 2 | Base models | Uniform | U | 22 | -27616.20 | 55276 |  |  |  |  |  |  |  |  |  |
| 3 | Base models | Triangular | T | 22 | -27542.35 | 55129 |  |  |  |  |  |  |  |  |  |
| 4 | Extended base models | Lognormal | LN | 22 | -27626.18 | 55296 |  |  |  |  |  |  |  |  |  |
| 5 | Extended base models | Log uniform | LU | 22 | -27650.24 | 55344 |  |  |  |  |  |  |  |  |  |
| 6 | Extended base models | Asymmetric triangular | AT | 25 | -27176.47 | 54403 |  |  |  |  |  |  |  |  |  |
| 7 | Flexible models | Fosgereau & Mabit ^2 | FM2 | 33 | -27284.56 | 54635 |  |  |  |  |  |  |  |  |  |
| 8 | Flexible models | Fosgereau & Mabit ^3 | FM3 | 44 | -27160.32 | 54409 |  |  |  |  |  |  |  |  |  |
| MA1 | Model averaging | Base models | MA(N,U,T) | 2 | -27322.41 | 54781 |  | 0.4132 | 0.2559 | 0.331 |  |  |  |  |  |
| MA2 | Model averaging | Base + extended | MA(N,U,T,LN,LU) | 4 | -27216.76 | 54662 |  | 0.2306 | 0.1343 | 0.2404 | 0.1858 | 0.2090 |  |  |  |
| MA3 | Model averaging | Base + extended + flexible | MA(N,U,T,LN,LU,AT,FM2,FM3) | 7 | -26873.87 | 54186 |  | 0. 0000 | 0.0000 | 0.0000 | 0.1037 | 0.000 | 0.2355 | 0.2005 | 0.4603 |

Table A4: results from willingness-to-pay space models on tobacco SP dataset. Pars – number of estimated parameters; LL – log-likelihood; AIC – Akaike Information Criterion. All models used 500 Modified Latin Hypercube Sampling (MLHS) draws. The model averaging shares are given in the right hand columns, e.g. “Share: at” is the share the model averaging allocated to the asymmetric triangular model.

Appendix 4: Calculation of model averaging weights

The model averaging weights are based on the estimates from the second stage regression, i.e. equation (15) in the main paper. Taking the MA3 model from table A4 as an example, we have the model estimates as,


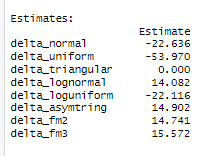


Then we simply these to shares using a logit formula. For example, for FM3, we have

$${share}_{fm3}= \frac{\exp\left( {delta}_{fm3} \right)}{\exp\left( {delta}_{n} \right)+\exp\left( {delta}_{u} \right)+\exp\left( {delta}_{t} \right)+\exp\left( {delta}_{ln} \right)+\exp\left( {delta}_{lu} \right)+\exp\left( {delta}_{at} \right)+\exp\left( {delta}_{fm2} \right)+\exp\left( {delta}_{fm3} \right)}= \frac{\exp\left( 15.572 \right)}{\exp\left( -22.636 \right)+\exp\left( -53.970 \right)+\exp\left( 0 \right)+\exp\left( 14.082 \right)+\exp\left( -22.116 \right)+\exp\left( 14.902 \right)+\exp\left( 14.741 \right)+\exp\left( 15.5732 \right)}=0.4603$$

It should be noted that the use of the logit formula can result in extreme estimates (e.g. -22.636 above) being found for models that are assigned a 0% share. To estimate standard errors of class shares, the above calculation must be repeated omitting models with a 0% share.

1. [↑](#footnote-ref-1)
2. Percentage of those eligible: excludes LC Models [↑](#footnote-ref-2)
